# Applying Radiomics to Predict Pathology of Postchemotherapy Retroperitoneal Nodal Masses in Germ Cell Tumors

**DOI:** 10.1200/CCI.18.00004

**Published:** 2018-05-11

**Authors:** Jeremy Lewin, Paul Dufort, Jaydeep Halankar, Martin O’Malley, Michael A.S. Jewett, Robert J. Hamilton, Abha Gupta, Armando Lorenzo, Jeffrey Traubici, Madhur Nayan, Ricardo Leão, Padraig Warde, Peter Chung, Lynn Anson Cartwright, Joan Sweet, Aaron R. Hansen, Ur Metser, Philippe L. Bedard

**Affiliations:** **Jeremy Lewin**, **Padraig Warde**, **Peter Chung**, **Lynn Anson Cartwright**, **Joan Sweet**, **Aaron R. Hansen**, and **Philippe L. Bedard**, Princess Margaret Cancer Centre; **Michael A.S. Jewett**, **Robert J. Hamilton**, **Madhur Nayan**, **Ricardo Leão**, **Aaron R. Hansen**, and **Philippe L. Bedard**, University of Toronto; **Paul Dufort**, **Jaydeep Halankar**, **Martin O’Malley**, and **Ur Metser**, University Health Network; and **Abha Gupta**, **Armando Lorenzo**, and **Jeffrey Traubici**, Hospital for Sick Children, Toronto, Ontario, Canada.

## Abstract

**Purpose:**

After chemotherapy, approximately 50% of patients with metastatic testicular germ cell tumors (GCTs) who undergo retroperitoneal lymph node dissections (RPNLDs) for residual masses have fibrosis. Radiomics uses image processing techniques to extract quantitative textures/features from regions of interest (ROIs) to train a classifier that predicts outcomes. We hypothesized that radiomics would identify patients with a high likelihood of fibrosis who may avoid RPLND.

**Patients and Methods:**

Patients with GCT who had an RPLND for nodal masses > 1 cm after first-line platinum chemotherapy were included. Preoperative contrast-enhanced axial computed tomography images of retroperitoneal ROIs were manually contoured. Radiomics features (n = 153) were used to train a radial basis function support vector machine classifier to discriminate between viable GCT/mature teratoma versus fibrosis. A nested 10-fold cross-validation protocol was used to determine classifier accuracy. Clinical variables/restricted size criteria were used to optimize the classifier.

**Results:**

Seventy-seven patients with 102 ROIs were analyzed (GCT, 21; teratoma, 41; fibrosis, 40). The discriminative accuracy of radiomics to identify GCT/teratoma versus fibrosis was 72 ± 2.2% (area under the curve [AUC], 0.74 ± 0.028); sensitivity was 56.2 ± 15.0%, and specificity was 81.9 ± 9.0% (*P* = .001). No major predictive differences were identified when data were restricted by varying maximal axial diameters (AUC range, 0.58 ± 0.05 to 0.74 ± 0.03). The prediction algorithm using clinical variables alone identified an AUC of 0.76. When these variables were added to the radiomics signature, the best performing classifier was identified when axial masses were limited to diameter < 2 cm (accuracy, 88.2 ± 4.4; AUC, 0.80 ± 0.05; *P* = .02).

**Conclusion:**

A predictive radiomics algorithm had a discriminative accuracy of 72% that improved to 88% when combined with clinical predictors. Additional independent validation is required to assess whether radiomics allows patients with a high predicted likelihood of fibrosis to avoid RPLND.

## INTRODUCTION

Multimodal treatment has dramatically increased the likelihood of cure in metastatic germ cell tumor (GCT), and the reduction of treatment morbidity is an important survivorship imperative. The standard of care for patients with metastatic nonseminoma GCT (NSGCT) is cisplatin-based chemotherapy followed by postchemotherapy retroperitoneal lymph node dissection (pcRPLND) for patients with residual nodal masses. These residual nodal masses occur in approximately 40% of patients treated with chemotherapy, and surgery is indicated if postchemotherapy axial nodal measurements are > 1 cm in the setting of marker plateau or normalization.^1^ The goal of surgery is to remove residual mature teratoma or viable GCT found in 30% to 40% and 5% to 10% of surgical specimens, respectively.^2,3^ Thus, approximately 50% of patients who undergo pcRPLND are found to have fibrosis/necrotic tissue alone. Because pcRPLND is associated with short- and long-term complications, such as time off from work, anesthetic risks, vascular complications, retrograde ejaculation, hernia, abdominal scarring, and chylous ascites,^4^ better discriminators are needed to differentiate between patients who require pcRPLND for detection of residual disease and those who do not.

Standard imaging with computed tomography (CT) or magnetic resonance imaging cannot reliably differentiate fibrosis from mature teratoma or viable GCT. Baseline clinical and pathologic factors have been investigated in their ability to detect residual active disease, including the presence of teratoma in the primary tumor, prechemotherapy tumor marker level, prechemotherapy nodal size, and interval size reduction during chemotherapy.^2,5-8^ However, no predictive algorithm is sufficiently sensitive to be used routinely in clinical practice beyond residual nodal size.^5,9^

The field of radiomics focuses on improving quantitative analysis of medical images by using automated high-throughput extraction.^10^ Texture analysis typically involves the accumulation of multidimensional histograms of image intensities. A large number of nonlinear metrics are computed from these distributions that measure properties like heterogeneity, directionality, and entropy and produce a large set of features that can subsequently be tested for accuracy in predicting treatment outcomes, even if the physiologic underpinnings are unknown. This approach is supported by research that demonstrates spatial variation of protein expression within tumors, which correlates to radiophenotypes in CT data.^11^ A radiomics approach to assessing retroperitoneal nodes is particularly well suited in GCT given that NSGCTs exhibit histomorphologic heterogeneity with various regions of teratoma, yolk sac, embryonal carcinoma, and choriocarcinoma that can be identified in both the primary tumor and the nodal metastasis. Thus, we hypothesized that radiomics would identify patients with a high likelihood of fibrosis who may avoid RPLND.

## PATIENT AND METHODS

The study aim was to determine whether texture features from postchemotherapy CT images can correctly discriminate between fibrosis and teratoma/GCT in patients who had undergone pcRPLND after first-line platinum-based chemotherapy for metastatic NSGCT.

### Patient Selection

This single-institution, retrospective study included patients diagnosed with NSGCT between January 1, 1995, and October 31, 2014, who had residual retroperitoneal masses after frontline cisplatin-based chemotherapy and who had undergone pcRPLND. The inclusion criteria were NSGCT histology with normalization or plateau of tumor markers after frontline cisplatin-based chemotherapy, residual nodal size > 1 cm on CT imaging measured through transverse axial dimension, and pathology from RPLND along with pre- and postchemotherapy CT imaging. Patients were excluded if they had a noncontrast CT scan or the investigators could not correlate the nodal mass on CT imaging with the pathology report. Patients were identified from an institutional testicular cancer database after research ethics board approval. Selected patients represented all three possible pathologic outcomes from their pcRPLND (teratoma, necrosis/fibrosis, viable GCT).

### Image Acquisition

Contrast-enhanced CT imaging of the abdomen and pelvis was performed with nonionic intravenous contrast (Appendix). Regions-of-interest (ROIs) were drawn circumferentially around each postchemotherapy residual nodal mass by two study team members (J.L., J.H.) after the residual nodal lesion was identified on the template RPLND pathology report and correlated with imaging.

### Texture Metrics

A set of 11 first-order and 142 second-order texture metrics were generated from each volume of interest (VOI), which comprised a set of two-dimensional ROIs that occupied a contiguous range of slices and overlapped from one slice to the next. The first-order metrics consisted of the 11 image intensity percentiles from each VOI and ranged from 0% (the minimum value) to 100% (the maximum value) with nine steps of 10% in between. These metrics provided a characterization of the one-dimensional image intensity histogram shape.

Before computing the 142 second-order texture metrics, the intensities within each VOI were binned into 32 equal-sized bins that spanned the range of image intensities between the first and 99th percentiles. The binning was conducted to minimize histogram noise when computing second-order texture metrics, whereas the use of image intensities between the first and 99th percentiles minimized the effect of outliers on the bin layout. The second-order texture features consisted of metrics from four classes computed from multidimensional histograms as follows: mean and range of the 13 Haralick texture features computed from the grayscale co-occurrence matrix^12^ taken over all 13 neighbor orientations^13^ on the three-dimensional lattice, five features based on the neighborhood gray tone difference matrix,^14^ 10 features from the gray-level run-length matrix,^15^ and the same 10 features from the gray-level size-zone matrix.^16^ Repetition of these groups at multiple resolutions produced the full set of 142 second order features.

### Machine Learning Algorithm

A nested 10-fold cross-validation protocol was used to determine classifier accuracy. Restricted axial and radial size criteria and clinically meaningful variables used in previous prediction algorithms were used to optimize the classifier (teratoma in primary, prechemotherapy tumor marker level, pre- and postchemotherapy mass size).^2,5-8^ Additional details of the radiomics algorithm are described in the Appendix.

### Statistical Analysis

Descriptive statistics were used to summarize baseline demographics. Machine learning protocol and other statistical analysis are described in the Appendix. Assessment of clinical variables to predict pathologic outcomes were analyzed through a published clinical nomogram.^7^ Maximum effective radii were defined as the radius of a sphere with the same nodal volume.

## RESULTS

### Included Population

**Fig 1.**
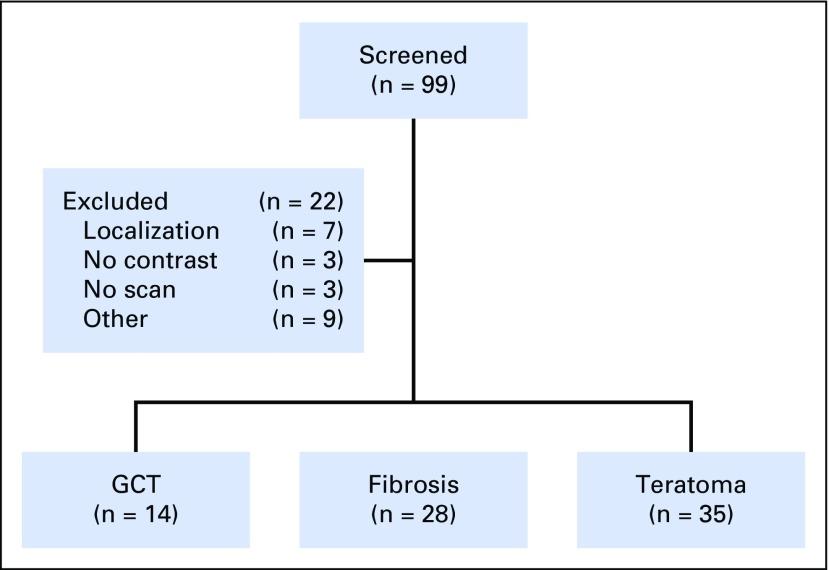
Patient flow diagram. GCT, germ cell tumor.

Through the institutional database, 322 patients with NSGCT underwent pcRPLND for lesions > 1 cm of whom 167 were identified between the January 1, 2007, and October 31, 2014, where routine depositing of imaging occurred on our institutional electronic server. From these patients, we specifically selected 99 with available pathology reports and imaging. Twenty-two patients were excluded because of difficulty in localization of nodal disease (n = 7), lack of contrast (n = 3), technical difficulties in retrieving imaging (n = 3), and other reasons (n = 9; Fig 1). The final cohort of 77 patients with 102 ROIs was used for analysis (fibrosis, n = 28 [40 ROIs]; teratoma, n = 35 [42 ROIs]; GCT, n = 14 [20 ROIs]). The existing literature has reported a rate of 5% to 10% chance of residual viable GCT.^2,3^ To develop a robust radiomics signature, we purposely included all patients with GCT from 2007 for analysis. Thus, residual GCTs in this cohort were over-represented and identified in 14 (18%) of 77 patients (Table 1). The characteristics of the patient population and ROIs are listed in Table 1. The mean size of the retroperitoneal masses were smaller for patients with fibrosis (46 mm) than for those with GCTs (63 mm) and teratomas (66 mm; *P* = .10).

**Table 1.**
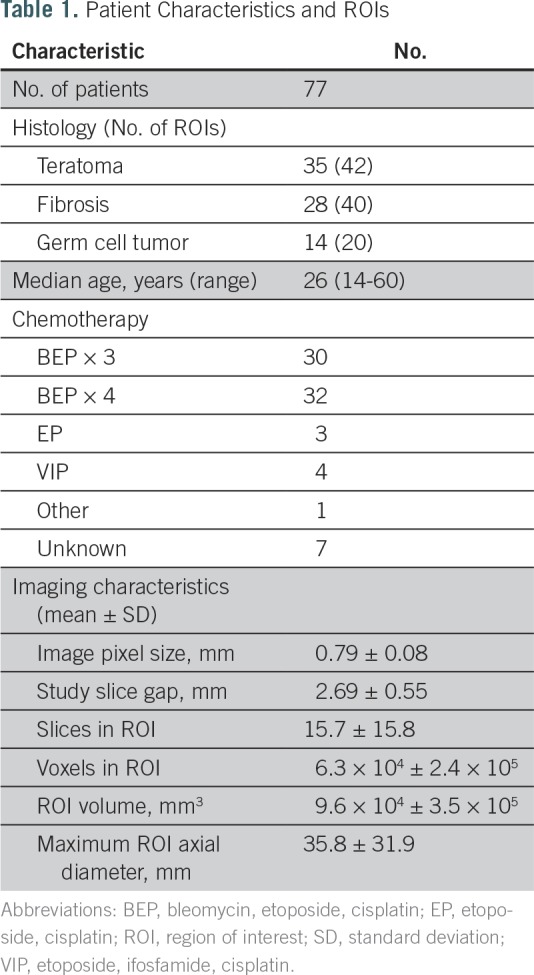
Patient Characteristics and ROIs

### Radiomics Signature

**Fig 2.**
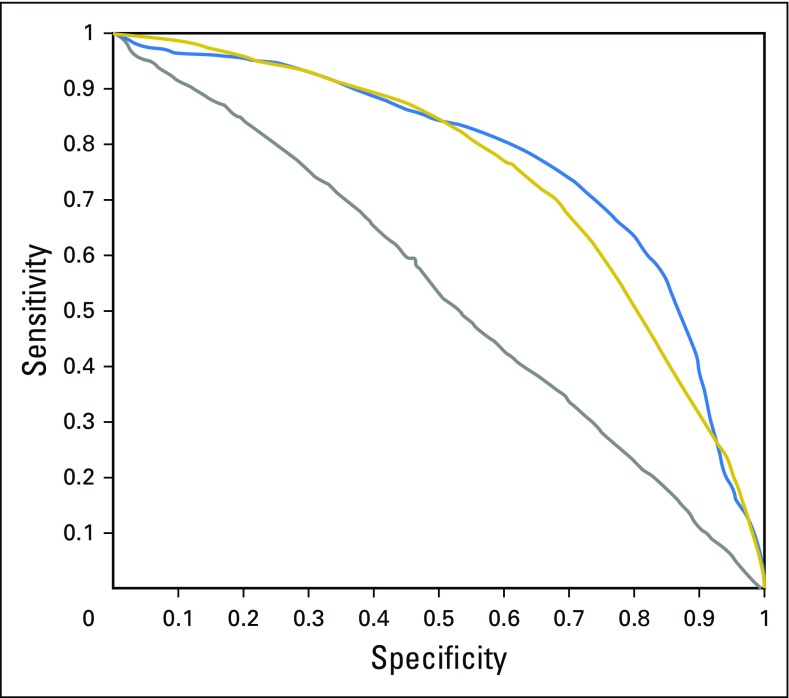
Receiver operating characteristic curves for radiomics classifier discrimination among three different binary configurations: teratoma (T) versus germ cell tumor (GCT)/fibrosis (F) (blue line); F versus T/GCT (gold line); GCT versus F/T (gray line). For F versus T/GCT, the classifier achieved a mean accuracy of 71.7 ± 2.2%, which corresponds to a sensitivity of 56.2 ± 15.0% and a specificity of 81.9 ± 9.0% with an area under the curve of 0.74 ± 0.028 (*P* = .001).

**Fig A1.**
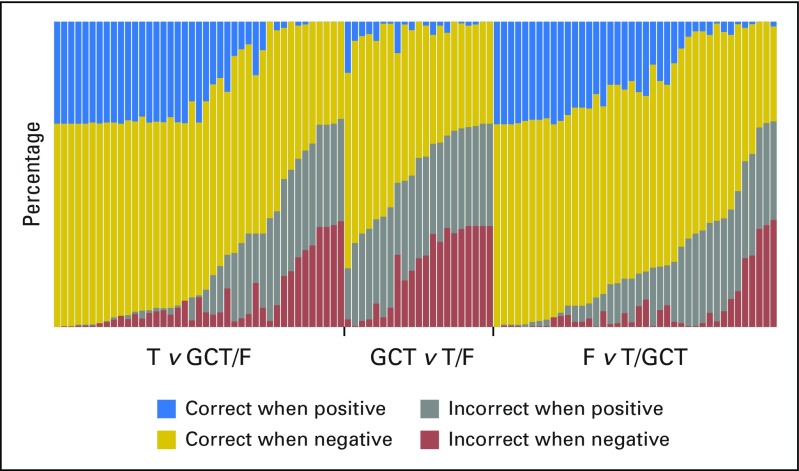
Illustration of classification accuracy for each of the 102 regions of interest (ROIs) over each of the three × 100 randomized trials, 100 for each of the three binary configurations. For the purposes of this figure, the two classes within each configuration are termed positive for the singlet class that contains only one type of lesion and negative for the doublet class that contains the two remaining lesion types grouped together. Each ROI is shown with the configuration for which the type was positive. Within each binary configuration, the ROIs are sorted in order of decreasing classification accuracy demonstrated by the support vector machine classifier from left to right. For each, the full set of three × 100 trials are represented by four colored bands. The blue bands represent the trials where the ROI was in the singlet (positive) class and classified correctly; the gold bands represent the trials where the ROI was in the doublet (negative) class and classified correctly; the gray bands represent the trials where the ROI was in the singlet class and classified incorrectly; and the red bands represent the trials where the ROI was in the doublet class and classified incorrectly. F, fibrosis; GCT, germ cell tumor; T, teratoma.

The receiver operating characteristic (ROC) curves for the three binary configurations that used the full 102 ROIs are shown in Figure 2. For the teratoma versus GCT/fibrosis configuration, the classifier achieved a mean accuracy of 75.4 ± 2.1% in 100 repetitions of the nested 10-fold cross-validation protocol, which corresponds to a sensitivity of 63.0 ± 8.6%, specificity of 83.8 ± 5.0%, and area under the curve (AUC) of 0.77 ± 0.023 (*P* = .001). For the GCT versus teratoma/fibrosis configuration, the classifier achieved a mean accuracy of 79.6 ± 0.4%, which corresponds to a sensitivity of 1.52 ± 3.76%, specificity of 99.9 ± 0.6%, and AUC of 0.53 ± 0.056 (*P* = .31). Finally, for the most clinically meaningful scenario of fibrosis versus teratoma/GCT configuration, the classifier achieved a mean accuracy of 71.7 ± 2.2%, which corresponds to a sensitivity of 56.2 ± 15.0%, specificity of 81.9 ± 9.0%, and AUC of 0.74 ± 0.028 (*P* = .001). In assessing the distribution of accuracies for individual nodes, we assessed the performance of the 102 ROIs over the 100 repetitions. Over the entire data set, only two nodes were classified correctly 100% of the time, and none were classified incorrectly more than two thirds of the time (Appendix Fig A1). Of the 41 nodes with teratoma, > 80% were classified correctly more than half of the time, whereas of the 40 nodes with fibrosis, 88% were classified correctly more than half of the time, but only 43% were classified correctly more than half of the time for the nodes with GCT.

Finally, we assessed which radiomics features were most useful in making predictions of pathologic outcomes. For the teratoma versus GCT/fibrosis classifier, 10 of the 153 features were statistically significantly correlated with the binary outcome after a false discovery rate correction to *q* = 0.05. All were first-order texture features, which reflects that teratoma possesses a lower distribution of CT densities. In contrast, 98 of the 153 features were significantly correlated with outcome for the fibrosis versus teratoma/GCT configuration, and of these, the top 10 were all second-order texture features that quantitated patterns of spatial heterogeneity in CT densities rather than gross changes in magnitudes.

### Restricted-Size Data Sets With and Without Clinical Variables

**Fig 3.**
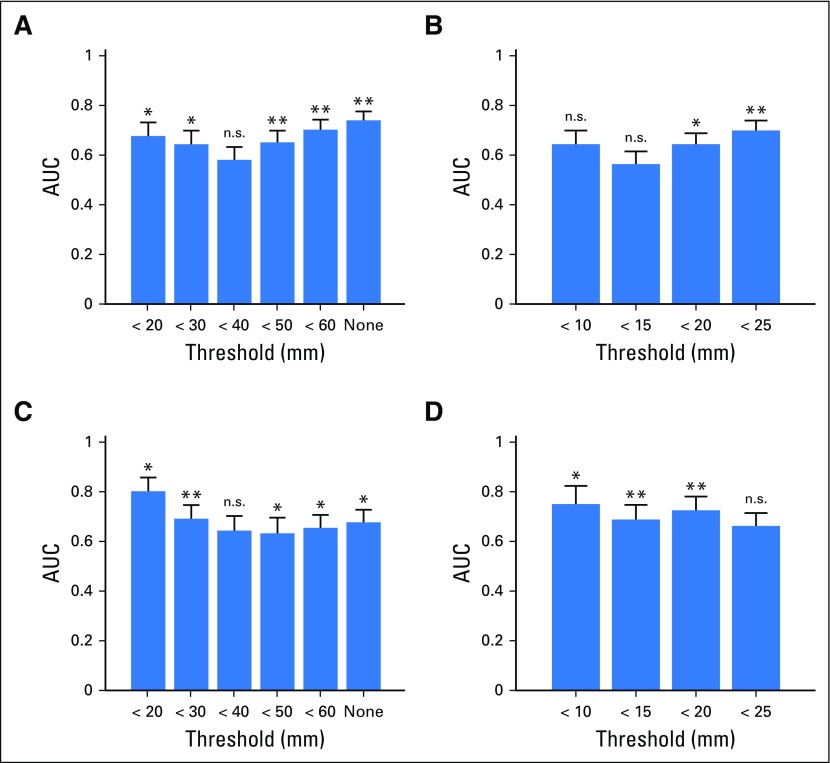
Bar plots of the area under the receiver operating characteristic curve for each of the four restricted cases: (A) restricted axial diameter, (B) restricted radial diameter, (C) restricted axial diameter with clinical variables, and (D) restricted radial diameter with clinical variables. The *y*-axis measures the area under the curve (AUC) from 0 to 1, whereas the *x*-axis indicates the restriction applied to the full data set. * significant at *P* ≤ .05; ** significant at *P* = .01. Fig 3A: < 40 mm, *P* = .14; Fig 3B: < 10 mm, *P* = .06; < 15 mm, *P* = .24; Fig 3C: < 40 mm, *P* = .11; Fig 3D: < 25 mm, *P* = .07.

We next aimed to assess whether the radiomics signature had different performance across varying size diameters with or without the inclusion of clinical variables. The performance of the classifier improved as the axial diameters increased, with an AUC of 0.58 at 40-mm axial cuts that increased to 0.74 with unrestricted size criteria (Table 2; Fig 3A). This was also identified when analyzing the data using maximum effective radii (maximum radius: 15 mm, AUC, 0.57; < 25 mm, AUC, 0.70; Fig 3B). However, the AUCs also increased for lower, more-restrictive thresholds, even as the size of the data sets was sharply reduced to < 20 mm in axial cuts (AUC, 0.67) and < 10 mm in maximum radius (AUC, 0.64; Figs 3A and B). Nonetheless, the improvement achieved by maximally restricting the data sets was not enough to overtake the best performance achieved with the largest, unrestricted data sets.

**Table 2.**
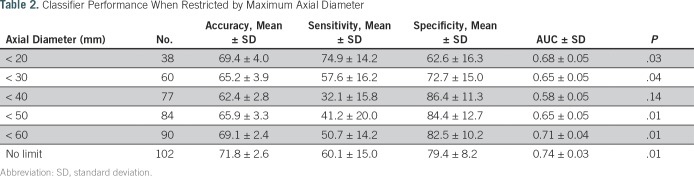
Classifier Performance When Restricted by Maximum Axial Diameter

We next analyzed the performance of a published clinical nomogram^7^ for patients with fully available clinical data (n = 40) and calculated the optimism-adjusted AUC using the bootstrapped procedure of Steyerberg et al.^17^ Using clinical variables alone (prechemotherapy tumor markers [alpha-fetoprotein, beta-human chorionic gonadotropin, lactate dehydrogenase]), residual mass size, percentage of mass shrinkage, and presence of teratoma elements in orchiectomy specimens), we calculated an AUC of 0.76. When these clinical variables were added to the radiomics classifier (Figs 3C and D), the AUC ranged from 0.63 when analyzing axial cutoffs of < 50 mm up to the highest observed AUC of 0.80 when the analysis was restricted to the smallest residual masses (axial nodal size < 20 mm).

## DISCUSSION

Although the role of pcRPLND for residual masses after platinum-based chemotherapy is well established, considerable debate continues with regard to which patients may safely avoid surgery. By using a postchemotherapy CT axial size cutoff of > 1 cm, approximately 50% of patients were found to have fibrosis alone at pcRPLND. When one limits pcRPLND to those with smaller lesions (eg, < 1 cm), the chance of viable GCT or mature teratoma is 11%.^5^ As a result, most guidelines endorse surveillance of small residual postchemotherapy lesions to reduce the burden of overtreatment.^18^ Kollmannsberger et al^1^ reported a cohort of 161 patients with residual lesions of < 1 cm who did not undergo pcRPLND, with only 10 relapses observed over a median follow-up of 52 months. Similarly, Ehrlich et al^19^ reported a cohort of patients with residual lesions < 1 cm from an Indiana University cohort managed expectantly; 12 (9%) of 141 patients experienced a relapse at a median of 15 years follow-up, and all but four successfully underwent salvage treatment. Nevertheless, others advocate for universal pcRPLND for all patients with nodal disease before platinum-based chemotherapy, irrespective of nodal size at postchemotherapy imaging, to reduce the risk of teratoma transformation, the potential toxicities of salvage chemotherapy, and the burden of surveillance imaging.^9^

Because imaging size criteria > 1 cm cannot reliably identify viable GCT or mature teratoma, several clinical prediction algorithms have been investigated.^2,5-8^ The most widely used algorithm includes six clinical variables (prechemotherapy tumor markers [alpha-fetoprotein, beta-human chorionic gonadotropin, lactate dehydrogenase], residual mass size, percentage of mass shrinkage, and the presence of teratoma elements in orchiectomy specimen).^7^ Despite a high discriminative accuracy (AUC range, 0.77 to 0.84),^7^ this model has not been universally adopted in clinical practice because of its complexity.^20^ With the use of radiomics, the current study identifies an AUC of 0.74 that improved to 0.80 in residual masses < 20 mm with the addition of clinical variables. Of note, the performance of our radiomics signature improved as the axial diameters increased, with an AUC of 0.58 at 40-mm axial cuts and 0.74 with unrestricted size criteria. This finding suggests that the modest discriminative accuracy may be related to the small sample size. However, even when limited to more-restrictive size criteria (and thus fewer patients), the data seem to show that the relationship between texture features and lesion type becomes cleaner and more deterministic when the focus of the analysis is restricted to smaller masses. Nonetheless, the improvement achieved by maximally restricting the data sets was not enough to overtake the best performance achieved with the largest, unrestricted data sets. Because we cannot entirely discount that the imbalance of nodal size in our data set among pathologic findings (fibrosis *v* teratoma *v* GCT) accounts for the variation of the classifier performance, a larger series across a range of residual nodal mass dimensions is required to optimize the radiomics signature.

There is great enthusiasm for the potential of quantitative image analysis to improve prediction of clinical outcomes.^21^ Radiomics models have been built to predict histologic subtypes,^22^ predict pathologic response to chemotherapy^23^ and chemoradiotherapy,^24^ and identify lymph node metastasis.^25^ Because NSGCT is known to exhibit histomorphologic heterogeneity, a quantitative radiomics approach theoretically should augment standard practice given its ability to provide information about spatial and temporal variability. In the current radiomics data set, the predictive ability to identify mature teratoma was based on exclusively first-order texture features, which reflect the frequently cystic appearance with low-density spatial regions on CT imaging. In contrast, the top 10 features that discriminate fibrosis from teratoma/GCT were all second-order texture features, which supports the role for mathematical prediction algorithms for patterns such as spatial heterogeneity, which are impossible to describe subjectively. A radiomics approach has been criticized for problems with reproducibility, especially with variations between machines and contrast timing; difficulty with segmentation, especially with lesions that display complex margins; and problems with externally validating radiomics models. In addition, our radiomics signature did not seem to improve established clinical nomograms.^7^ Thus, although the approach is novel, additional independent validation that addresses these criticisms is required to determine whether the signature can be optimized to make it clinically usable. Because the cancer radiomics field is still in its infancy, large-scale clinical application still requires data processing uniformity, harmonization of informatics infrastructure, and standardization with regard to the reporting of radiomics features.

Given the variable success of clinical and radiomics prediction methods, molecular markers hold promise with regard to their ability to detect residual disease. Molecular studies have demonstrated that levels of serum microRNAs correlate with stage of disease^26^ and reduction in response to treatment in patients with metastatic disease.^27^ Early reports support this by having demonstrated that plasma levels of miR-371 correlate with the presence of active germ cell malignancy in surgical specimens, with miR-371 being undetectable in any samples with no viable tumor (zero of nine) and overexpressed in 11 of 12 samples with viable GCT.^28^ Future studies are required to investigate whether these molecular markers replace or complement a radiomics approach or replace standard prediction models to safely avoid pcRPLNDs in patients with low predicted risk of residual teratoma or viable GCT.

In the setting of a highly curable disease, overwhelming clinical evidence is needed to change practice; thus, the application of radiomics or molecular algorithms with high negative predictive value will not eliminate the need for ongoing surveillance imaging for patients at low risk of residual mature teratoma. In such patients, more-frequent abdominopelvic CT surveillance imaging is required compared with patients who are treated with pcRPLND. Concerns exist about an increased risk of second malignancy for patients who undergo frequent CT imaging, such as those with clinical stage I NSGCT managed with active surveillance versus primary RPLND.^29^ Thus, patients must be counseled about the risk of surveillance imaging versus overtreatment with pcRPLND for fibrosis alone, similar to discussions that occur in the stage 1 setting.^30^

The current findings have a number of important limitations. First, this retrospective, single-institution analysis used a selected patient sample that was validated with internal bootstrapping without an external validation cohort. Second, the analysis was restricted to patients with postchemotherapy residual retroperitoneal nodal masses. Whether these findings can be translated to residual masses at other anatomic sites, such as liver or lung, where surrounding normal parenchymal changes induced by chemotherapy may be increased and thus may limit radiomics prediction algorithms is unclear. Third, radiomics is a technically challenging and time-consuming approach that is unlikely to be clinically deliverable at places outside larger tertiary centers without improvements in automated contouring of ROIs. Fourth, detection of microscopic residual viable GCT in an otherwise fibrotic or necrotic large lymph node mass may be impossible to detect by a radiomics approach because of the limited resolution of a CT scan. Finally, there was no central pathology review, although all patient cases were reported in the same department by expert genitourinary pathologists and quality assurance procedures.

In summary, we developed a predictive radiomics algorithm that had an overall discriminative accuracy of 72% that improved to 88% when combined with clinical predictors. Additional independent validation is required to assess whether radiomics, in conjunction with standard clinical predictors, can identify patients with a high predicted likelihood of fibrosis to avoid pcRPLND.

## Appendix

### Computed Tomography Imaging

Contrast-enhanced computed tomography (CT) imaging of the abdomen and pelvis was performed with a 64-multidetector CT scanner (Aquilion 64; Toshiba Medical Systems, Otawara, Tochigi, Japan) with an individual detector width of 0.5 mm, a 0.5-second gantry rotation time, and a table speed of 53 mm/rotation. The following scan parameters were used: detector collimation, 0.5 mm × 64; reconstruction slice thickness, 5 mm; increment, 2.5 mm; and tube current determined by automated tube modulation, 120 kVp. Coronal reformations (reconstruction thickness, 3 mm; increment, 3 mm) were available for review. The scan was performed after injection of nonionic intravenous contrast material (iohexol, 30 mg iodine/mL [Omnipaque 300; GE Healthcare, Chicago, IL) with a power injector at a dose of 2 mL/kg up to a maximum of 200 mL at a rate of 3 mL/s with a 60-second delay.

### Correlation Between Nodal Size and Pathology Outcomes

Preliminary analyses indicated the presence of outliers in the distributions of the maximum nodal size. Because this can reduce the power of parametric tests and bias effect size estimates, Wilcox (Introduction to Robust Estimation and Hypothesis Testing [ed 4], Academic Press, 2017) has recommended the use of 20% trimmed means and Winsorized variances for statistical inference. We used the percentile-*t* bootstrap version (Keselman et al: Psychol Sci 15:47-51, 2004) of Yuen’s (Biometrika 61:165-170, 1974) two-sample *t* test to compare fibrosis and nonfibrosis (teratoma and germ cell tumor [GCT]) groups. As a corresponding measure of effect size, we used the percentile bootstrap version (Keselman et al: Psychol Methods 13:110-129, 2008) of the robust Cohen’s *d* (dt) of Algina et al (Psychol Methods 10:317-328, 2005) on the basis of 20% trimmed means and Winsorized variances. For all bootstrapped statistics, 2,000 resamples were drawn with replacement from the original data. The WRS package of R (https://github.com/nicebread/WRS) was used for this series of analyses.

### Machine Learning

All machine learning was carried out using the support vector machine (SVM) algorithm with a radial basis function kernel implemented using the LibSVM software library (Chang et al: ACM Trans Intell Syst Technol 2:27, 2011) and accessed through an associated MATLAB (MathWorks, Natick, MA) interface. The goal of the machine learning was to train a classifier to predict whether each volume of interest contained a teratoma, GCT, or fibrotic region on the basis of the texture features extracted from the voxels within the volume of interest. Because the SVM algorithm is only capable of discriminating between two classes, the training algorithm was applied to the data in three binary configurations: teratoma versus GCT and fibrosis; GCT versus teratoma and fibrosis; and fibrosis versus teratoma and GCT.

For each SVM training run, it was necessary to tune three hyperparameters that governed the behavior of the classifier. The first hyperparameter pertained to feature selection. An F-statistic approach (Chen et al: Feature Extraction 207:315-324, 2006) was used to rank the 153 input texture features in order of their association with the response classification. A tunable hyperparameter representing the fraction of the most highly associated features to keep was then applied to select the features that were used. The second hyperparameter was the standard cost parameter common to all flavors of SVM, whereas the third was the width of the gaussian curve that makes up the radial basis function kernel.

A nested cross-validation scheme was used to tune the three hyperparameters while keeping the assessment of accuracy completely independent. In each of 100 iterations of the outer loop, 10-fold cross-validation was used to hold out 10% of the data for testing, whereas the remaining 90% was passed to the inner loop. Within the inner loop, an additional 10-fold cross-validation protocol was used for each point in a three-dimensional grid that covered a range of fractions of the best features to retain, values of the SVM cost parameter, and values of the radial basis function width. The inner loop cross-validation result was recorded for each grid point searched, and at the conclusion of the inner loop, the best performing triple of hyperparameters was used to train a classifier using all of the inner loop data. This classifier was then applied to classify the held-out data from the outer loop, and the results were recorded as the classifier’s accuracy.

### Receiver Operating Characteristic and Other Post Hoc Analyses

An SVM classifier does not produce a dichotomous binary classification as its output but rather as a single, continuous number on the real line. Only when a threshold is applied to it is it transformed into a classification. This makes it possible to adjust the threshold to trade off sensitivity and specificity, thus creating a receiver operating characteristic (ROC) curve. Furthermore, repeating the outer loop of the nested cross-validation protocol 100 times yields 100 such numbers for each tumor, which results in a more fine-grained ROC curve than would be possible with no repetitions. Each of the 100 numbers for a particular tumor represented an instance in which it was held out during cross-validation with a different 10% of the data and allowed for a more robust characterization of the accuracy for that patient. The mean ± standard deviation accuracies reported are the best from each of the 100 ROC curves, and the sensitivities and specificities were taken from the point on each curve where the best accuracy was found. The ROC curves displayed in the figures were generated by combining all of the trial data into a single curve. In addition to producing the ROC curve, we examined the percentage of the 100 repetitions in which each patient case was classified or misclassified to determine whether particular patient cases were consistently misclassified or whether the errors were evenly spread among all patient cases.

### Analyses of Restricted Data Sets

An additional set of analyses was planned to explore the possibility that the inclusion of tumors of vastly different sizes in the same data set may reduce the effectiveness of the machine learning approach. Specifically, tumors at opposite ends of the size spectrum may in fact be genotypically and/or phenotypically different, as evidenced by either significantly different growth rates or different ages, one or both of which must account for the size differences. Tumors that are genotypically and/or phenotypically different may embody different radiomics signatures, which makes it difficult for the machine learning algorithm to arrive at a definitive discriminative pattern.

To assess this possibility, two additional rounds of machine learning analysis were undertaken in which the full set of patient cases was restricted to include only tumors within a certain size range. In the first, the maximum cross-sectional diameter of each tumor in the axial plane was measured across all axial slices that contained the tumor, and a series of six thresholds (20 mm, 30 mm, 40 mm, 50 mm, 60 mm, and no limit) was applied to exclude tumors with diameters greater than each threshold. The spacing of these thresholds was chosen so that enough new patient cases were introduced after each increase in the threshold for one to reasonably expect that the results could change. In the second round, the same principle was used, but in this case, the thresholds represented the effective radius of the tumor, defined as the radius of a sphere with the same volume as the tumor. Four effective radius thresholds were used (10 mm, 15 mm, 20 mm, 25 mm), again, because each new step caused a sufficient number of new patient cases to be included.

In this set of experiments, only a single binary classification was examined that discriminated fibrosis from GCT and teratoma combined (the most clinically relevant case). Aside from this and the restriction of the data sets by maximum axial diameter or effective radius, all other aspects of these machine learning trials were identical to the protocol described previously.

### Augmentation With Clinical Variables

Because this work was originally undertaken to examine the possible use of radiomics signatures for tumor classification and to compare the results with existing techniques on the basis of clinical variables, some of the experiments described here were repeated with the set of radiomics variables augmented with the best known clinical variables. The goal was to determine whether the information contained in the radiomics signatures was redundant with or complementary to the information embodied in the clinical variables. For each restricted analysis described in the previous section, the analysis was repeated with the radiomics data set augmented with key clinical variables (teratoma in primary, prechemotherapy tumor marker level, pre- and postchemotherapy mass size). Patient cases with any of the variables missing were excluded, which brought the total number before the application of size restrictions down to 61.
